# Esophageal Schwannoma—Systematic Review of Clinicopathologic Factors and Treatment

**DOI:** 10.3390/jcm15082862

**Published:** 2026-04-09

**Authors:** Rashad Khazen, Raneem Bader, George Asfour, Barak Bar-Zakai, Guy Pines, Harbi Khalayleh

**Affiliations:** 1 Department of Surgery, Kaplan Medical Center, Rehovot 7661041, Israel; rashad.khazen@gmail.com (R.K.); asfour-george23@hotmail.com (G.A.); barakba@clalit.org.il (B.B.-Z.); 2 Faculty of Medicine, Hebrew University of Jerusalem, Jerusalem 9112102, Israel; 3 Department of Surgery, Samson Assuta Ashdod University Hospital, Ashdod 7747629, Israel; raneembader1995@gmail.com; 4 Faculty of Health and Science, Ben-Gurion University of the Negev, Beer Sheva 8410501, Israel; 5 Department of Thoracic and Esophageal Surgery, Kaplan Medical Center, Rehovot 7661041, Israel

**Keywords:** esophageal, schwannoma, enucleation, esophagectomy

## Abstract

**Background**: Esophageal schwannomas are extremely rare, benign mesenchymal tumors originating from the nerve sheath tissues of autonomic nerves, accounting for less than 2% of all esophageal tumors. This systematic review aims to provide a detailed analysis of esophageal schwannomas (ESs), focusing on tumor characteristics, diagnostic methods, and treatment options. **Methods**: A systematic search of English literature databases, including ScienceDirect, Springer, PubMed, and Google Scholar, was conducted up to 2023. The keywords used were ‘esophageal schwannoma,’ ‘gastrointestinal schwannoma,’ ‘esophageal neurinoma,’ and ‘esophageal neurilemoma.’ Studies were reviewed for patient demographics, clinical presentation, diagnostic methods, tumor characteristics, and management options. **Results**: A total of 370 articles met the inclusion criteria, with 80 articles (89 cases) included in the final analysis. The mean age of patients was 51.8 years, with a female predominance (73%). Most cases were reported from East Asia (60.7%). Most (71%) patients presented with dysphagia, and 12% were asymptomatic. Preoperative diagnosis often involved CT scans (75.28%), upper endoscopy (73.03%), and EUS (49.4%). Tumors averaged 77.86 mm in size as per CT, MRI and PET-CT, with the upper esophagus being the most common location (55.55%). Surgical resection was the primary treatment, with enucleation being the most frequent procedure (58.9%). The prognosis was generally excellent, with no reported recurrences during follow-up periods. **Conclusions**: Esophageal schwannomas are extremely rare. Surgical resection remains the treatment of choice, with a high success rate and excellent prognosis. Further studies are needed to standardize diagnostic and treatment protocols for these rare tumors.

## 1. Introduction

Schwannomas are slow-growing benign mesenchymal tumors that originate from submucosal proliferating nerve sheath tissue of autonomic nerves, belonging to Auerbach’s plexus or Meissner’s plexus [1,2,3]. Schwannomas are more often found in the stomach and duodenum [3,4] and are rarely found in the esophagus, and account for less than 2% of all esophagus tumors [1,2,3,4,5,6,7]. Gastrointestinal schwannomas account for 0.4 to 1.0% of all submucosal tumors of the gastrointestinal tract [4].

Given their rarity and often asymptomatic nature, preoperative diagnosis might be challenging [2] with no established diagnosis protocol.

Imaging methods such as computed tomography (CT) cannot distinguish the ES from other esophageal tumors [2]. Therefore, a final diagnosis could only be made by a surgical or an EUS-guided biopsy [2,4].

Due to the rarity of ES, there is no standardized surgical approach; however, the preferred management is a minimally invasive approach, mainly enucleation [2,4,8].

In this systematic review, we analyze all English-published studies regarding esophageal schwannomas and analyze tumor characteristics, diagnostic approaches, and treatment options.

## 2. Materials and Methods

### 2.1. Search Strategy

This systematic review was performed under the Preferred Reporting Items for Systematic Reviews and Meta-Analyses [PRISMA] 2020 guidelines. The review was not registered in PROSPERO, and no formal review protocol was prepared prior to commencement. Literature databases including “ScienceDirect”, “Springer”, “PubMed” and “google scholar” were systematically searched up to 2023, using the following keywords: “esophageal schwannoma”, “gastrointestinal schwannoma”, “esophageal neurinoma” and “esophageal neurilemoma”.

This review follows the systematic review methodology as defined by the PRISMA 2020 framework (Table S1): a pre-specified, multi-database search strategy with explicit eligibility criteria, independent dual screening, standardized data extraction, and formal quality appraisal using the JBI Critical Appraisal Checklist for Case Reports. The evidence base consists of case reports and case series, which is the predominant—and often the only available—study design for rare conditions such as esophageal schwannoma. This precludes a formal PICO Comparison component and inferential statistical synthesis; the findings are therefore descriptive in nature, and the overall certainty of evidence is rated as very low under the GRADE framework, as detailed in the Limitations Section. This study design and methodology are consistent with published systematic reviews in this specific field [9].

### 2.2. Study Selection

All the studies were reviewed, and the following information was extracted: patients’ characteristics (age, gender, nationality), disease presentation and symptoms, diagnosis methods, tumor characteristics, and management options. Only English-language studies were included.

### 2.3. Data Extraction

Titles and abstracts of studies were independently assessed and reviewed by two reviewers. The full texts of potentially relevant papers were retrieved and rescreened. The data were extracted using an Excel datasheet (Microsoft Corp, Redmond, WA, USA) and this included authors, year of publication, and the study location. We also extracted demographic characteristics of the patients, symptoms, work-up, diagnostic tools, treatment strategies and outcomes.

### 2.4. Statistical Analysis

All the statistical tests were performed using EXCEL.

Categorical data are presented as frequencies and percentages, while continuous data are represented as means ± standard deviations (SDs) or medians, depending on data distribution.

### 2.5. Risk of Bias Assessment

The methodological quality of all included studies was appraised using the JBI Critical Appraisal Checklist for Case Reports (JBI, 2020). This validated eight-item tool assesses the following domains: (1) clear identification of the patient’s demographic information; (2) clear description of the patient’s history; (3) clear description of the presenting concern; (4) appropriate diagnostic tests and results; (5) adequate description of the intervention; (6) clear description of the post-intervention clinical condition; (7) identification of any adverse events; and (8) whether the case report provides actionable learning. Each item was rated as “yes,” “no,” or “unclear/not applicable.” Given the inherent nature of case reports and case series—which constitute the entirety of the evidence base for this rare condition—a formal risk of bias score was assigned to each included study; however, no study was excluded solely on the basis of quality assessment. Two reviewers independently conducted the appraisal, and discrepancies were resolved by consensus.

### 2.6. Reporting Bias and Certainty of Evidence Assessment

Given that all included studies are case reports or small case series, formal assessment of reporting bias using funnel plots or statistical methods was not feasible. Instead, potential reporting biases—including publication bias (favoring unusual or positive outcomes), selection bias, and language bias (English-only inclusion)—were acknowledged qualitatively. The certainty of the overall body of evidence was assessed in accordance with GRADE (Grading of Recommendations Assessment, Development and Evaluation) principles. As all data derive from observational case reports without a comparison group, the evidence is rated as “very low” certainty by default under the GRADE framework. This designation reflects the inherent limitations of the study design rather than flaws in reporting, and is consistent with the rarity of the condition under review.

## 3. Results

**Figure 1:**
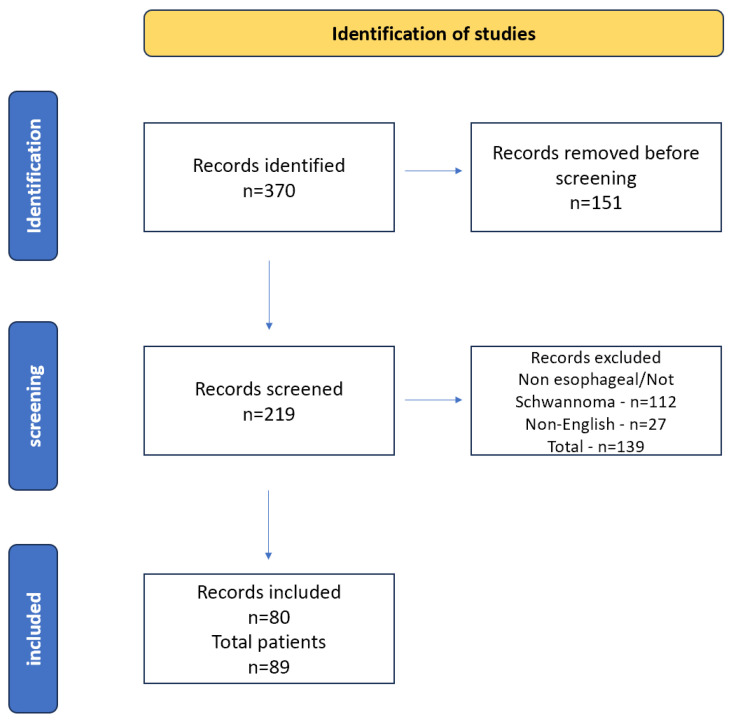
Study flow card.

A total of 370 articles met the inclusion criteria, and 290 were excluded because they were either duplicated, about non-esophageal schwannoma tumors, or they were non-English publications. After exclusion, a total of 80 articles were included in the study (Figure 1). Table 1 presents the included studies and detailed characteristics.

### 3.1. Demographical Characteristics

A total of 89 patients were included in the study. Mean patient age was 51.8 years (11–78). Most patients were females (73%, *n* = 65) and 22 (24.27%) were males; in two cases (2.24%) this was not reported. Most patients (54/89, 60.7%) were from Asia (East and South), with the majority from Japan (44.44%, 24/54) and China (31.48%, 17/54). Most non-East Asian countries (33.71%, *n* = 30) included the United States (8/30, 26.67%) and the United Kingdom (3/29 10.34%) (Table 1 and Table 2).

### 3.2. Clinical Presentation

The clinical presentation was reported in 83 of the 89 cases (93.3%). Ten patients (10/83, 12%) were asymptomatic, and their tumor was an incidental finding. Most (73/83, 88%) patients were symptomatic. Reported symptoms included dysphagia (*n* = 59,71.1%), dyspnea or stridor (*n* = 25, 30.12%), heartburn (*n* = 12, 14.46%), gastroesophageal reflux disease (*n* = 4, 4.82%), weight loss (*n* = 7, 8.43%), and a cervical mass (*n* = 6, 7.2%).

Less frequent symptoms included: abdominal distention (3.6%), fatigue (2.4%), dysphonia (2.4%), hemoptysis (1.2%), shoulder pain (1.2%), chronic cough (1.2%), and paresthesia of left hand (1.2%).

The duration of symptoms ranged from 0.5 to 72 months, with an average of 15.9 months (Table 3).

### 3.3. Preoperative Diagnosis and Tumor Size

Preoperative diagnostic strategy was reported in 92% of the cases (82/89). The most frequent diagnostic modalities that were used to identify schwannomas were computed tomography scans (CT) (75.28%), upper endoscopy (73.03%), and endoscopic ultrasound (EUS) (49.4%). Other modalities were Positron emission tomography (PET) (16.85%), magnetic resonance imaging (MRI) (13.48%), and bronchoscopy (10.11%) (Table 4).

The tumor size in EUS or endoscopy was reported in 23 cases (25.8%), while tumor size as in CT/MRI/PETCT/Barium study was documented in 51 cases (57.3%). Intraoperative tumor size was reported in 69 cases (77.6%).

The average size of schwannoma as per endoscopy or EUS was 79.68 mm (range: 5–97 mm), and as per CT, MRI, PET-CT, and barium study, was 77.86 mm (range: 15–170 mm). Among operated patients, the mean size of resected schwannoma was 71.58 mm (range: 2.7–200 mm).

### 3.4. Location

Tumor location was reported in 61% of the cases (54/89). The most common location of schwannoma was in the upper third of the esophagus (*n* = 30, 55.55%). The rest were distributed equally between middle esophagus and the lower third (22.22% and 22.22%, respectively).

### 3.5. Tumor Markers

Tumor markers were reported in 88% of the cases (78/89). Tumor markers (carcinoembryonic antigen (CEA), Carbohydrate Antigen 19-9 (CA19-9), Squamous Cell Carcinoma Antigen (SCC), or Sialyl SSEA-1 antigen (SLX)) were reported in 13 cases. Two cases (15.4%) had positive markers, while 11 (84.62%) had negative markers.

### 3.6. Management

Management strategies were reported in 82 of the 89 cases.

Seventy-three cases (82%) underwent surgical resection, and 7 patients (7.87%) underwent endoscopic resection. One patient underwent hybrid surgical and endoscopic management (1.12%), and one patient was treated conservatively (1.12%).

Among patients who underwent surgical resection, 46 patients (63.01%) underwent open surgery, 11 (15.07%) underwent video-assisted thoracoscopic surgery (VATS), two patients underwent laparoscopic surgery (2.73%), and one patient underwent robotic surgery (1.4%).

The most frequent surgical intervention was enucleation (58.9%, *n* = 43). Ten (13.7%) patients underwent subtotal esophagectomy, 10 (13.7%) patients underwent total esophagectomy, 9 (12.33%) patients underwent En-bloc resection, and one patient (1.4%) underwent Distal Esophagectomy with Gastrectomy (Table 5).

### 3.7. Histology

The histology of 79 (88.8%) cases was reported. This figure refers to postoperative histological confirmation following surgical or endoscopic resection; in the remaining 11.2% of cases, no formal histopathological report was retrievable from the source publication. It should be noted that all patients ultimately received a histological diagnosis of schwannoma—either confirmed preoperatively via EUS-guided fine-needle aspiration biopsy (EUS-FNAB) or, more commonly, postoperatively after resection. Sixty-six cases (74.16%) were reported as benign, while 7 (7.78%) were reported as malignant. Four cases (4.49%) were reported to have a plexiform (ancient) schwannoma and 2 (2.25%) had melanotic schwannoma.

### 3.8. Lymph Nodes

Preoperative lymph node status was reported in 21 cases (23.6%). Of these, 5 (23.81%) were positive, and 16 (76.19%) were negative.

Postoperative status of lymph nodes was reported for 10 cases. Two (2.25%) had positive involvement of lymph nodes (metastasis), while 8 (8.99%) had negative involvement of lymph nodes.

### 3.9. Immunohistochemical Tumor Markers

Thirty-six immunohistochemical tumor markers were examined and reported. Table 6 shows the details of immunohistochemical examination.

### 3.10. Post-Interventional Course and Complications

Post-Interventional course was reported in 65 cases. Fifty-nine patients (66.29%) had an uneventful post-interventional course, while 6 (6.74%) suffered from complications. Complications included fever (*n* = 1), wound infection (*n* = 1), leak (*n* = 1), dysphonia or laryngeal nerve palsy (*n* = 2), and leak with pneumonia (*n* = 1). Only one case of the 43 cases that underwent enucleation had postoperative leak with pneumonia.

### 3.11. Prognosis and Recurrence

The mean follow-up period was 26 months [3–72 months]. The recurrence status of the disease was reported in 60 cases (67%). No recurrence was reported. It is important to note that follow-up data were unavailable for a substantial proportion of cases, and the absence of reported recurrences may therefore reflect incomplete reporting rather than a uniformly favorable oncological outcome. This limitation should be considered when interpreting prognosis data.

In 24 cases recurrence was not reported or not relevant due to death of the patient (24/89, 26.96%).

One patient died after 5 months due to distant metastasis of malignant schwannoma.

## 4. Discussion

In this study we have performed a systematic review of the literature on the presentation, diagnosis and management of esophageal schwannomas [1,2,3,4,5,6,7,8,9,10,11,12,13,14,15,16,17,18,19,20,21,22,23,24,25,26,27,28,29,30,31,32,33,34,35,36,37,38,39,40,41,42,43,44,45,46,47,48,49,50,51,52,53,54,55,56,57,58,59,60,61,62,63,64,65,66,67,68,69,70,71,72,73,74,75,76,77,78,79,80]. The least common location of schwannomas in the GI tract is in the esophagus [2,6,9] and esophageal schwannomas represent less than 2% of all esophageal tumors [1,2,4]. Esophageal schwannomas are predominant in Asian ethnicity [2], and are more common in females, in the 5th-6th decade of life [2,5,6,8]. In our review, more than half of the cases (73%) were females, with a mean age of 51.8 years old, with more than half of the cases (60.7%) of East Asian patients. Most esophageal schwannomas are found incidentally and are asymptomatic [1,2,5]. Symptomatology depends on the mass size and location [2]. The most common symptom is dysphagia [2,5]. Other symptoms can be dyspnea, chest pain, or epigastric pain [2,5]. In our review, only ten cases (12%) had no symptoms, and their tumor was an incidental finding, while 73 (82%) were symptomatic. The most common symptom was dysphagia, followed by dyspnea, heartburn, weight loss, feeling of a neck mass, gastroesophageal reflux disease, abdominal distention, fatigue, dysphonia, shoulder pain, chronic cough, and paresthesia of the left hand.

Tumor size was found to have significant variability, with different diagnostic modalities—EUS (5–97 mm), in CT/MRI/PET-CT/barium study (15–170 mm), and intra-operatively (2.7. to 200 mm). The mean sizes were 79.68 mm, 77.86 mm and 71.58 mm, respectively. Notably, the average size differs between EUS and other imaging modalities and intra-operatively, likely because EUS can detect smaller lesions.

Preoperative diagnosis of esophageal schwannomas can be challenging due to their featureless and nonspecific characteristics in imaging studies such as CT or MRI [1,2,6]. Biopsies taken during endoscopy may not be useful, since schwannomas involve submucosa and muscularis propria [4]. The diagnostic modality of choice for schwannomas and submucosal tumors is endoscopic ultrasound-guided fine-needle aspiration biopsy (EUS-guided FNAB) [1,2,4,8]. EUS-FNAB has a diagnostic accuracy of 85.2% for submucosal tumors. Diagnostic strategy depends on local resources and expertise, but combination of CT and EUS with or without FNAB provides the most significant information.

Immunohistochemical markers play a vital role in the diagnosis of schwannomas and in ruling out different diagnoses [2]. Leiomyomas are considered the most common benign esophageal tumors, and they express smooth muscle markers, unlike schwannomas [2,5]. Also, GISTs overexpress CD117(c-kit), but schwannomas do not [2,5]. Positive S-100 supports the diagnosis of schwannomas [6,7,10]. Likewise, the positivity of leu-7 and lack of smooth muscle markers such as Actin, Desmin, and CD-34, support the diagnosis of schwannomas [1,5,7]. Therefore, confirmation of diagnosis requires pathologic examination with further immuno-staining studies after surgical resection [5]. In our study, all reported cases had positive S-100 proteins. Other positive markers were Vimentin, NSE, and Glial Fibrillary Acidic protein. Negative markers were c-KIT/CD117, CD34, SMA, Desmin, DOG-1, Actin and GFAP.

The treatment of choice for esophageal schwannomas is either surgical or endoscopic resection [5] depending on tumor size and local expertise. Surgical enucleation and esophagectomy are considered the standard of care in the management of esophageal schwannomas [1,2,5,11]. If the mass is well-demarcated with no signs of deep muscular invasion in the EUS, an endoscopic approach could be considered [2]. In our review most patients underwent surgical resection (82%), mainly enucleation (58.9%).

The choice of therapeutic approach in the included cases was driven by a combination of clinical and tumor-related factors. Enucleation—the most frequently performed procedure—was generally preferred for well-circumscribed tumors confined to the muscularis propria with no evidence of malignancy, lymph node involvement, or deep infiltration on preoperative imaging. Esophagectomy (subtotal or total) was typically reserved for larger tumors (many exceeding 60–100 mm), those in the distal esophagus, or cases with malignant features or suspected lymph node involvement on CT or EUS. Endoscopic resection was selected predominantly for smaller, superficially located lesions at centers with advanced endoscopic expertise. The absence of lymph node involvement (reported as negative in 76.2% of cases with available preoperative data) frequently supported a conservative, organ-preserving approach. In summary, the therapeutic decision was individualized based on tumor size, location, depth of invasion, preoperative imaging findings, and the availability of local surgical or endoscopic expertise.

Benign schwannomas do have an excellent prognosis [6]. All patients for whom recurrence data were available had no recurrence in the follow-up period. Only one patient died 5 months after resection due to distant metastasis.

### Limitations

This review has several important limitations that must be acknowledged. First, the review was not prospectively registered in PROSPERO, and no formal protocol was prepared prior to data extraction, which limits reproducibility and introduces the potential for post hoc methodological decisions. Second, the search was restricted to four electronic databases (ScienceDirect, Springer, PubMed, and Google Scholar) and limited to English-language publications. This approach, while systematic, may have missed relevant studies published in other languages or indexed in databases such as Embase, CINAHL, or regional repositories, introducing a language bias and information bias. Third, the evidence base consists entirely of individual case reports and small case series, which are inherently susceptible to selection bias (favoring surgically managed or unusual presentations), publication bias (favoring positive outcomes), and reporting bias (incomplete or selective data disclosure). As a result, the certainty of all conclusions derived from this review is very low according to the GRADE framework. Fourth, formal quality appraisal using the JBI Critical Appraisal Checklist for Case Reports revealed variable completeness of reporting across studies, particularly regarding follow-up duration, management rationale, and histological workup. Fifth, follow-up data were unavailable for a substantial number of cases (29 of 89 patients had no reported follow-up), meaning the statement of “no reported recurrences” should be interpreted cautiously, as it may reflect missing data rather than a uniformly favorable oncological outcome. Finally, owing to the purely descriptive nature of the included studies and the absence of a comparison group, no inferential statistical analyses were possible, and causal conclusions regarding treatment efficacy cannot be drawn.

## 5. Conclusions

While esophageal schwannomas are rare, awareness of their clinical and pathological features is essential for accurate diagnosis and effective management. Minimally invasive enucleation is safe and associated with a low complication rate. Future research should focus on refining diagnostic techniques and exploring minimally invasive surgical options to further improve patient outcomes.

## Figures and Tables

**Table 1:** Characteristics of previous reported cases.

	Author	Year	Country	Patients (N=)	Gender	Resection Method	Follow Up (Months)	Histology
1	Zackria R [1]	2021	US	1	F	N/R	N/R	N/R
2	Matteo MV [2]	2020	Italy	1	M	Subtotal esophagectomy	N/R	plexiform “ancient” schwannoma
3	T. Manger [3]	2000	Germany	1	F	Subtotal esophagectomy	48	malignant schwannoma
4	Watanabe T [4]	2016	Japan	1	F	Total esophagectomy	N/R	benign esophageal schwannoma
5	Zhang Y [5]	2018	China	1	F	En-bloc resection	50	benign esophageal schwannoma
6	Basoglu A [6]	2006	Turkey	1	F	Subtotal esophagectomy	40	malignant schwannoma
7	Park BJ [7]	2006	Peru	1	F	Total esophagectomy	12	benign esophageal schwannoma
8	Łochowski MP [8]	2016	Poland	1	N/R	Enucleation	N/R	benign esophageal schwannoma
9	Morales-Maza J [9]	2019	Mexico	1	F	N/R	N/R	N/R
10	Degheili JA [10]	2019	Lebanon	1	F	En-bloc resection	N/R	benign esophageal schwannoma
11	Khalayleh H [11]	2021	Israel	1	F	Enucleation	N/R	benign esophageal schwannoma
12	Zhu L [12]	2019	China	1	F	Subtotal esophagectomy	24	benign esophageal schwannoma
13	Wu CX [13]	2020	China	1	F	Total esophagectomy	36	benign esophageal schwannoma
14	Souza LCA [14]	2019	Brazil	1	M	Total esophagectomy	N/R	benign esophageal schwannoma
15	Moro K [15]	2017	Japan	1	M	Enucleation	N/R	benign esophageal schwannoma
16	Iwata [16]	2018	Japan	1	F	Enucleation	60	benign esophageal schwannoma
17	Trindade AJ [17]	2018	US	1	M	EMR	N/R	benign esophageal schwannoma
18	Saito R [18]	2000	Japan	1	F	Enucleation	10	benign esophageal schwannoma
19	Iwata H [19]	1993	Japan	1	F	Enucleation	28	benign esophageal schwannoma
20	Li B [20]	2020	China	3	1:M 2:F 3:M	1:EMR 2:ESE 3:ESE	54 48 24	benign esophageal schwannoma
21	Kobayashi N [21]	2000	Japan	1	F	Enucleation	32	benign esophageal schwannoma
22	Hsu SD [22]	2003	Taiwan	1	M	Enucleation	16	benign esophageal schwannoma
23	Liu D [23]	2015	China	1	F	Enucleation	N/R	benign esophageal schwannoma
24	Gao ZY [24]	2021	China	1	F	ESD	N/R	benign esophageal schwannoma
25	Kitada M [25]	2013	China	1	F	Subtotal esophagectomy	N/R	benign esophageal schwannoma
26	Sanchez-Garcia Ramos E [26]	2019	Mexico	1	F	En-bloc resection	N/R	benign esophageal schwannoma
27	An B [27]	2019	Japan	1	F	Enucleation	N/R	benign esophageal schwannoma
28	Tokunaga T [28]	2007	Japan	1	F	Enucleation	N/R	benign esophageal schwannoma
29	Shimamura Y [29]	2016	Canada	1	M	EMR	N/R	benign esophageal schwannoma
30	Chen HC [30]	2006	Taiwan	1	F	Enucleation	12	benign esophageal schwannoma
31	Onodera Y [31]	2017	Japan	1	F	Enucleation	N/R	benign esophageal schwannoma
32	Choo SS [32]	2011	US	1	F	Enucleation	N/R	N/R
33	Favre-Rizzo J [33]	2016	Spain	1	F	Total esophagectomy	6	benign esophageal schwannoma
34	Ahn D [34]	2017	South Korea	1	F	Enucleation	12	benign esophageal schwannoma
35	Ferrante M [35]	2014	US	1	F	Enucleation	N/R	benign esophageal schwannoma
36	Kassis ES [36]	2012	US	1	M	Total esophagectomy	10	N/R
37	Tomono A [37]	2015	Japan	1	F	Subtotal esophagectomy	8	benign esophageal schwannoma
38	Mizuguchi S [38]	2008	Japan	1	F	Enucleation	5	benign esophageal schwannoma
39	Wang YL [39]	2015	China	2	F	Enucleation	N/R	benign esophageal schwannoma
40	Gu MJ [40]	2014	South Korea	1	M	Enucleation	27	benign esophageal schwannoma
41	Ngaage DL [41]	2002	United Kingdom	1	F	N/R	N/R	melanotic schwanoma
42	Retrosi G [42]	2009	Italy	1	F	En-bloc resection	N/R	plexiform “ancient” schwannoma
43	Mishra B [43]	2016	India	1	F	Total esophagectomy	18	malignant schwannoma
44	Jeon HW [44]	2014	South Korea	2	1 M 1 F	1 Enucleation 2 En-bloc resection	7 6	benign esophageal schwannoma
45	Murase K [45]	2001	Japan	1	F	Enucleation	16	malignant schwannoma
46	Hu Z [46]	2017	China	1	F	Enucleation	N/R	benign esophageal schwannoma
47	Yoon HY [47]	2008	South Korea	1	M	Enucleation	24	N/R
48	Makino T [48]	2013	Japan	1	M	Enucleation	24	benign esophageal schwannoma
49	Kozak K [49]	2015	Poland	1	F	Enucleation	N/R	benign esophageal schwannoma
50	Matsuki A [50]	2009	Japan	1	F	Enucleation	14	benign esophageal schwannoma
51	Ohno M [51]	2000	Japan	1	F	Enucleation	N/R	benign esophageal schwannoma
52	Wang S [52]	2011	China	1	F	Enucleation	12	malignant schwannoma
53	Chen X [53]	2016	China	3	1 M 2 F	Enucleation	N/R	benign esophageal schwannoma
54	George M [54]	2009	India	1	F	Enucleation	18	plexiform “ancient” schwannoma
55	Vinhais SN [55]	2004	India	1	F	Enucleation	36	benign esophageal schwannoma
56	Prévot S [56]	1999	France	1	F	Enucleation	29	benign esophageal schwannoma
57	Mavroeidis VK [57]	2017	Greece	1	F	Distal Esophagectomy with Gastrectomy	5	malignant schwannoma
58	Tomizawa K [58]	2020	Japan	1	F	Total esophagectomy	16	malignant schwannoma
59	Dutta R [59]	2009	India	1	F	Total esophagectomy		benign esophageal schwannoma
60	Kwon MS [60]	2002	South Korea	1	F	Enucleation	9	N/R
61	Arai T [61]	1994	Japan	2	F	N/R	N/R	benign esophageal schwannoma
62	Eberlein TJ [62]	1992	US	1	M	Total esophagectomy	60	benign esophageal schwannoma
63	Naus PJ [63]	2001	US	1	M	Polypectomy	24	N/R
64	Liu T [64]	2013	China	1	F	Subtotal esophagectomy	N/R	benign esophageal schwannoma
65	Cokelaere K [65]	2000	Belgium	1	F	Enucleation	8	plexiform “ancient” schwannoma
66	R M Brown [66]	2002	United Kingdom	1	F	Subtotal esophagectomy	46	melanotic schwanoma
67	T Nakatsu [67]	2011	Japan	2	F	Enucleation	32 30	benign esophageal schwannoma
68	S Sato [68]	2012	Japan	1	F	Enucleation	N/R	benign esophageal schwannoma
69	Hsu HH [69]	2002	Taiwan	1	F	N/R	6	benign esophageal schwannoma
70	Hughes SC [70]	2003	United Kingdom	1	M	En-bloc resection	36	benign esophageal schwannoma
71	Horiuchi A [71]	2004	Japan	1	M	Total esophagectomy	20	benign esophageal schwannoma
72	K. Sato [72]	2005	Japan	1	F	En-bloc resection	N/R	benign esophageal schwannoma
73	K. Dalci [73]	2014	Turkey	1	F	Enucleation	3	benign esophageal schwannoma
74	E. Toyama [74]	2008	Japan	1	F	Enucleation	N/R	benign esophageal schwannoma
75	Sachdeva V [75]	2008	India	1	F	Subtotal esophagectomy	N/R	benign esophageal schwannoma
76	Al-Kahil M [76]	2026	US	1	M	Enucleation	3	benign esophageal schwannoma
77	T. Okai [77]	2003	N/R	2	1 F 1 M	N/R	N/R	N/R
78	B. H. A. von Rahden [78]	2004	N/R	1	N/R	N/R	N/R	benign esophageal schwannoma
79	Li Poa [79]	2019	N/R	1	F	En-bloc resection	N/R	benign esophageal schwannoma
80	Alberto Mitsuo [80]	2016	N/R	1	M	En-bloc resection	N/R	benign esophageal schwannoma

**Table 2:** Patient clinical characteristics.

Patients Characteristics		N = 89 (100%)
**Age (in Years)**	Range	11–78
	Average	51.8
	Not reported	1
**Gender:**	Male	22 (24.27%)
	Female	65 (73%)
	Not reported	2
**Nationality:**		
Asian (East and South)		54 (54/89, 60.7%)
	Chinese	17 (17/54, 31.48%)
	Japan	24 (24/54, 44.44%)
	India	4 (4/54, 7.41%)
	South Korea	6 (6/54, 11.11%)
	Taiwan	3 (3/54, 5.56%)
Not East Asian		30 (33.71%)
	United States	8 (8/30, 26.67%)
	Italian	2 (2/29, 6.9%)
	Poland	2 (2/29, 6.9%)
	Mexico	2 (2/29, 6.9%)
	Brazil	1 (1/29, 3.45%)
	United Kingdom	3 (3/29, 10.34%)
	Turkey	2 (2/29, 6.9%)
	Spain	1 (1/29, 3.45%)
	Portugal	1 (1/29, 3.45%)
	Canada	1 (1/29, 3.45%)
	Peru	1 (1/29, 3.45%)
	Israel	1 (1/29, 3.45%)
	Lebanon	1 (1/29, 3.45%)
	Belgium	1 (1/29, 3.45%)
	France	1 (1/29, 3.45%)
	Greece	1 (1/29, 3.45%)
	Germany	1 (1/29, 3.45%)
	Not reported	5 (5/89, 5.6%)

**Table 3:** Clinical presentation.

Presentation		*n* (%)
**Initial Symptoms** **Reported = 83 (83/89, 93.3%)**	Dysphagia	59 (59/83, 71.1%)
	Dyspnea/stridor	25 (25/83, 30.12%)
	Retrosternal pain/heartburn	12 (12/83, 14.46%)
	Incidentally	10 (10/83, 12%)
	Gastroesophageal Reflux Disease	4 (4/83, 4.82%)
	Weight loss	7 (7/83, 8.43%)
	Neck mass	6 (6/83, 7.2%)
	Abdominal distension	3 (3/83, 3.6%)
	Fatigue	2 (2/83, 2.4%)
	Dysphonia	2 (2/83, 2.4%)
	Hemoptysis	1 (1/83, 1.2%)
	Shoulder pain	1 (1/83, 1.2%)
	Chronic cough	1 (1/83, 1.2%)
	Paresthesia of the left hand	1 (1/83, 1.2%)
	Other	6 (6/83, 7.2%)
	Not reported	4 (4/83, 4.82%)
**Period of Symptoms (in Months):**	Range	0.5–72
	Average	15.9
	Not reported	38 (38/89, 42.7%)

**Table 4:** Preoperative diagnostic modalities.

Preoperative Diagnostic of the Reported 82 Patients (82/89, 92.1%) *		*n* (%)
Endoscopic ultrasonography	No	38 (42.7%)
	Yes	44 (49.4%)
Computed tomography scan	No	15 (16.85%)
	Yes	67 (75.28%)
Endoscopy	No	17 (19.1%)
	Yes	65 (73.03%)
Positron emission tomography	No	67 (75.28%)
	Yes	15 (16.85%)
Magnetic resonance imaging	No	70 (78.65%)
	Yes	12 (13.48%)
Barium/gastrographin esophagogram	No	52 (58.43%)
	Yes	30 (33.71%)
Bronchoscopy	No	73 (82.02%)
	Yes	9 (10.11%)

* In 7/89 (7.87%) the preoperative modalities were not reported.

**Table 5:** Management of the reported cases.

Management	*n* (%)	Type		
**Reported cases**	82 (82/89, 92.13%)			
**Operative**	73 (73/89, 82%)	Type of operative management		***n*** **(%)**
			**Operative**	73 (73/89, 82%)
		Reported cases	**Type of operation:**	60
			Open	46 (46/73, 63.01%)
			Video-Assisted Thoracoscopic Surgery	11 (11/73, 15.07%)
			Robotic	1 (1/73, 1.4%)
			Laparoscopic	2 (2/73, 2.73%)
			Not reported/not relevant	29 (29/73, 39.73%)
		Reported cases	**Operation’s name**	73 (73/89, 82%)
			Subtotal esophagectomy	10 (10/73, 13.7%)
			En-bloc resection with primary closure by Esophagoplasty	9 (9/73, 12.33%)
			Distal Esophagectomy with Gastrectomy	1 (1/73, 1.4%)
			Total esophagectomy	10 (10/73, 13.7%)
			Enucleation	43 (43/73, 58.9%)
			Not reported/not relevant	16 (16/73, 8.22%)
**Endoscopic**	7 (7/89, 7.87%)	Type of endoscopic management		***n*** **(%)**
			**Endoscopic**	7 (7/68, 10.2%)
			Endoscopic mucosal resection	2 (2/7, 28.5%)
			Submucosal tunneling endoscopic resection	2 (2/7, 28.5%)
			Endoscopic Submucosal Dissection	1 (1/7, 14.2%)
			Endoscopic Submucosal Excavation	1 (1/7, 14.2%)
			Endoscopic polypectomy	1 (1/7, 14.2%)
**Operative and endoscopic**	1 (1/89, 1.12%)			
**Conservative**	1 (1/89, 1.12%)			
**Not reported**	7 (7/89, 7.87%)			

**Table 6:** Immunohistochemical examination.

Immunohistochemical Tumor Markers	Reported % = *n*/All (89)	Positive % = *n*/Reported	Negative % = *n*/Reported	Not Reported % = *n*/All (89)
S-100 protein	78 (87.64%)	78 (100%)	0 (0%)	11 (12.36%)
c-KIT/CD117 (tyrosine-protein kinase KIT, CD117/cluster of differentiation 117)	51(57.3%)	0 (0%)	51 (100%)	37 (41.57%)
CD34 (cluster of differentiation 34)	48 (53.93%)	2 (4.17%)	46 (95.83%)	41 (46.07%)
SMA (Smooth Muscle Actin)	43 (48.31%)	0 (0%)	43 (100%)	46 (51.69%)
Desmin	36 (40.45%)	0 (0%)	36 (100%)	53 (59.55%)
DOG-1 (Discovered on GIST-1)	14 (15.73%)	0 (0%)	14 (100%)	75 (84.27%)
vimentin	15 (16.85%)	14 (93.33%)	1 (6.67%)	74 (83.14%)
NSE (Neuron-specific enolase)	12 (13.48%)	9 (75%)	3 (25%)	77 (86.51%)
Actin	11 (12.36%)	0 (0%)	11 (100%)	78 (87.64%)
GFAP (Glial Fibrillary Acidic Protein)	7 (7.87%)	3 (42.87%)	4 (57.14%)	82 (92.13%)
Ki-67 (Kiel-67)	7 (7.87%)	4 (57.14%)	3 (42.87%)	82 (92.13%)
Lea-7 (Late Embryogenesis Abundant)	4 (4.49%)	3 (75%)	1 (25%)	85 (95.5%)
EMA (Epithelial membrane antigen)	6 (6.74%)	1 (1.67%)	5 (83.33%)	83 (93.26%)
Cytokeratin	5(5.62%)	0 (0%)	5 (100%)	84 (94.38%)
Neurofilaments	5 (5.62%)	0 (0%)	5 (100%)	84 (94.38%)
Synaptophysin	1 (1.12%)	0 (0%)	1 (100%)	5 (5.62%)
KIT/alpha KIT	6 (6.74%)	0 (0%)	6 (100%)	83 (93.26%)
PG9.5 protein (Protein Gene Product 9.5)	3 (3.34%)	3 (100%)	0 (0%)	86 (96.63%)
ALK (Anaplastic lymphoma kinase)	3 (3.34%)	0 (0%)	3 (100%)	86 (96.63%)
HMB45 (Human Melanoma Black 45)	4 (4.49%)	1 (25%)	3 (75%)	85 (95.5%)
MelanA (melanoma)	3 (3.34%)	2 (66.67%)	1 (33.33%)	86 (96.63%)
CD68 (cluster of differentiation 68)	2 (2.25%)	1 (50%)	1 (50%)	87 (97.75%)
AE1/AE3 (pankeratin AE13)	4(4.49%)	0 (0%)	4 (100%)	85 (95.5%)
Nestin	2 (2.25%)	2 (100%)	0 (0%)	87 (97.75%)
Pankeratin	2 (2.25%)	0 (0%)	2 (100%)	87 (97.75%)
Laminin	1 (1.12%)	1 (100%)	0 (0%)	88 (98.88%)
P53 (phosphoprotein p53)	2 (2.25%)	0 (0%)	2 (100%)	87 (97.75%)
Inhibin	2 (2.25%)	0 (0%)	2 (100%)	87 (97.75%)
Chromogranin	1 (1.12%)	0 (0%)	1 (100%)	88 (98.88%)
H-caldesmon	1 (1.12%)	0 (0%)	1 (100%)	88 (98.88%)
MSA (mammary serum antigen)	1 (1.12%)	0 (0%)	1 (100%)	88 (98.88%)
bcl-2 (cell lymphoma 2)	1 (1.12%)	1 (100%)	0 (0%)	88 (98.88%)
SOX-10 (Sry-related HMg-Box gene 10)	2 (2.25%)	2 (100%)	0 (0%)	87 (97.75%)
DPG-1 (diphosphoglycerate)	2 (2.25%)	0 (0%)	2 (100%)	87 (97.75%)
Calponin	2 (2.25%)	0 (0%)	2 (100%)	87 (97.75%)
COD1 (cone dystrophy)	1 (1.12%)	0 (0%)	1 (100%)	88 (98.88%)

Eleven cases were not reported at all.

## Data Availability

No new data were created or analyzed in this study. All data reviewed are from previously published peer-reviewed publications and are summarized in Table 1, Table 2, Table 3, Table 4, Table 5 and Table 6 within the manuscript.

## Supplementary Materials

The following supporting information can be downloaded at https://www.mdpi.com/article/10.3390/jcm15082862/s1, Table S1: PRISMA 2020 Checklist.

## Abbreviations

The following abbreviations are used in this manuscript:

N/R	Not reported
ALK	Anaplastic Lymphoma Kinase
CA19-9	Carbohydrate-Antigen 19-9
CEA	Carcinoembryonic Antigen
CT	Computed Tomography
DOG-1	Discovered on GIST-1
EMA	Epithelial Membrane Antigen
EMR	Endoscopic Mucosal Resection
ES	Esophageal Schwannoma
ESD	Endoscopic Submucosal Dissection
ESE	Endoscopic Submucosal Excision
EUS	Endoscopic Ultrasound
EUS-FNAB	Endoscopic Ultrasound-guided Fine-Needle Aspiration Biopsy
FNAB	Fine-Needle Aspiration Biopsy
GFAP	Glial Fibrillary Acidic Protein
GI	Gastrointestinal
GIST	Gastrointestinal Stromal Tumor
GRADE	Grading of Recommendations Assessment, Development and Evaluation
HMB45	Human Melanoma Black 45
JBI	Joanna Briggs Institute
Ki-67	Kiel-67 (proliferation marker)
Leu-7	CD57/Late Embryogenesis Abundant protein
MRI	Magnetic Resonance Imaging
NSE	Neuron-Specific Enolase
PET	Positron Emission Tomography
PICO	Population, Intervention, Comparison, Outcome
PRISMA	Preferred Reporting Items for Systematic Reviews and Meta-Analyses
PROSPERO	International Prospective Register of Systematic Reviews
S-100	S-100 Protein (calcium-binding protein)
SCC	Squamous Cell Carcinoma Antigen
SLX	Sialyl SSEA-1 Antigen
SMA	Smooth Muscle Actin
SOX-10	SRY-related HMG-Box gene 10
VATS	Video-Assisted Thoracoscopic Surgery
